# Steroidogenic Factor 1 in the Ventromedial Nucleus of the Hypothalamus Regulates Age-Dependent Obesity

**DOI:** 10.1371/journal.pone.0162352

**Published:** 2016-09-06

**Authors:** Ann W. Kinyua, Dong Joo Yang, Inik Chang, Ki Woo Kim

**Affiliations:** 1 Departments of Pharmacology and Global Medical Science, Wonju College of Medicine, Yonsei University, Wonju, 26426, Republic of Korea; 2 Department of Oral Biology, Yonsei University College of Dentistry, Seoul, 03722, Republic of Korea; 3 Division of Hypothalamic Research, Department of Internal Medicine and Department of Pharmacology, UT Southwestern Medical Center, Dallas, TX, 75390, United States of America; Sungkyunkwan University, REPUBLIC OF KOREA

## Abstract

The ventromedial nucleus of the hypothalamus (VMH) is important for the regulation of whole body energy homeostasis and lesions in the VMH are reported to result in massive weight gain. The nuclear receptor steroidogenic factor 1 (SF-1) is a known VMH marker as it is exclusively expressed in the VMH region of the brain. SF-1 plays a critical role not only in the development of VMH but also in its physiological functions. In this study, we generated prenatal VMH-specific SF-1 KO mice and investigated age-dependent energy homeostasis regulation by SF-1. Deletion of SF-1 in the VMH resulted in dysregulated insulin and leptin homeostasis and late onset obesity due to increased food intake under normal chow and high fat diet conditions. In addition, SF-1 ablation was accompanied by a marked reduction in energy expenditure and physical activity and this effect was significantly pronounced in the aged mice. Taken together, our data indicates that SF-1 is a key component in the VMH-mediated regulation of energy homeostasis and implies that SF-1 plays a protective role against metabolic stressors including aging and high fat diet.

## Introduction

Steroidogenic factor 1 (SF-1) is a nuclear receptor expressed in the adrenal glands, gonads, anterior pituitary, and ventromedial nucleus of the hypothalamus (VMH) [1]. SF-1 is vital not only for the development of the VMH but also for its physiological functions [2, 3]. The VMH is an important hypothalamic nucleus critical for regulating feeding and maintaining whole body energy homeostasis. Lesions in the VMH alter feeding behavior and have been associated with hyperphagia and development of obesity [4]. Similarly, the VMH functions as a nutrient sensor and has been shown to respond to declining nutritional conditions such as hypoglycemia by inhibiting insulin production and stimulating glucagon and catecholamines release [5]. In addition, the expression of leptin receptors in the VMH depicts the importance of this nucleus in responding to leptin induced feeding, metabolism and energy balance regulation [6, 7].

Previous studies have implicated SF-1 and SF-1 neurons as important metabolic regulators in the VMH [8–10]. Whereas whole body SF-1 knockout is lethal due to adrenal insufficiency, corticosterone injection and adrenal transplants rescues the SF-1 knockout mice but is accompanied by severe obesity indicating that SF-1 deficiency alters energy metabolism [11]. Mice lacking leptin receptor in SF-1 expressing neurons specifically in the VMH exhibited metabolic syndrome features including obesity, elevated insulin and leptin levels and impaired glucose tolerance [8, 12]. Moreover, insulin receptor knockout mice in SF-1 neurons showed improved glucose metabolism and were protected against high fat diet induced leptin resistance and weight gain and deletion of the transcription factor FoxO1 in SF-1 neurons showed increased energy expenditure and improved insulin sensitivity that exhibited an overall lean phenotype [9, 13]. In addition, mice lacking vesicular glutamate transporter 2 (Vglut2) in SF-1 neurons exhibited a modest increase in body weight when exposed to high fat diet (HFD) and also developed hypoglycemia during fasting due to impaired induction of the glucose raising hormone glucagon and the gluconeogenic enzymes PEPCK and G6Pase [14]. Although these studies point to the importance of SF-1 neurons in the maintenance of normal energy homeostasis in the VMH, the mechanism regulating this energy metabolism by SF-1 is not well understood. Further the role of SF-1 in modulating age-dependent energy homeostasis under different nutritional conditions has not been well elucidated.

We previously reported that postnatal deletion of SF-1 in the VMH leads to high fat diet induced obesity due to impaired thermogenesis and blunted leptin signaling [15]. In the present study, we sought to investigate the role of SF-1 in age-dependent metabolic regulation in the VMH by generating different aged SF-1 KO mice. Here, we report that deletion of SF-1 in the VMH leads to late onset of obesity that is largely associated with increased food intake, blunted energy expenditure and reduced physical activity in the aged KO mice. Additionally, the SF-1 KO mice showed dysregulated insulin and leptin homeostasis and a reduction in the activity of the brown adipose tissue (BAT) as indicated by decreased expression of uncoupling protein 1 (UCP1). Further, the expresion of Vglut2 in the VMH was blunted in the SF-1 KO mice. Taken together, our data underpins the importance of SF-1 in the VMH-mediated energy balance regulation and implies that SF-1 is critical for protection against age-dependent and diet induced metabolic disorders.

## Materials and Methods

### Animal Care

Mice were maintained at room temperature (22–24°C) with a 12 hour dark/light cycle with the lights switched on at 6am and switched off at 6pm. Mice were fed either normal chow (NC; Teklad mouse/rat diet, no. 7001; 4.25% kcal from fat, 3.82 kcal/g) or high fat diet (Research Diet no. D12331; 58% kcal from fat, 26% from sucrose, 5.56 kcal/g) with free access to water. The VMH specific SF-1 KO mice were generated by crossing SF-1^F/F^ female mice with SF-1 ^+/-^ male mice expressing nestin-cre as described previously [16]. For metabolic cage studies, the mice were allowed to acclimatize in the metabolic chambers for 6 days and provided with food and water *ad libitum*. O_2_ consumption, CO_2_ production and heat generation were monitored for 5 days after acclimatization and the rearing and ambulatory movements monitored with infrared beams. All the above animal experiments were approved by the Institutional Animal Care and Use Committee at The University of Texas-Southwestern Medical Center and Yonsei University Wonju College of Medicine.

### Glucose Tolerance Test (GTT)

Mice were fasted overnight for 18h and provided with water *ad libitum*. The following morning, the mice were housed in individual cages and stabilized for one hour. The mice were administered with 1g/kg glucose dissolved in saline and blood was collected from the tail nick at 0, 15, 30, 60, and 120 minutes. The glucose level was measured using the Contour TS glucometer (Ascensia Diabetes Care, BRK, UK).

### Insulin Tolerance Test (ITT)

Mice were acclimatized in individual cages and fasted for one hour and provided with water *ad libitum*. Basal glucose level was measured after which 1U/kg insulin was administered intraperitoneally and the blood glucose level monitored at 0, 15, 30, 60, and 120 minutes using the Contour TS glucometer (Ascensia Diabetes Care, BRK, UK).

### Glucose, Insulin and Leptin Measurements

The plasma glucose, insulin and leptin levels were assayed in fed condition and after 24h fasting condition. For glucose measurement, blood was collected from the tail nick and assayed through the glucose oxidase method using Contour TS glucometer (Ascensia Diabetes Care, BRK, UK). Insulin and leptin measurements were conducted at the Mouse Metabolic Phenotyping Core Laboratory at the University of Texas Southwestern Medical Centre at Dallas.

### RNA Isolation and Quantitative Real Time PCR

Mice were decapitated after deep anesthesia with i.p. injection of 2,2,2 Tribromoethanol (Avertin) at a dosage of 250 mg/kg. Total RNA was isolated from brown adipose tissue (BAT) using Trizol reagent (Invitrogen, CA, USA) and cDNA was synthesized with the SuperScript First-Strand Synthesis System (Invitrogen, CA, USA) following the manufacturer’s protocol. Q-PCR primers for TaqMan method include 18S (ABI; Hs99999901_s1), UCP1 (ABI; Mm01244861_m1), β3AR (ABI: Mm02601819_g1) and PPARγ (Mm01184322_m1).

### *In Situ* Hybridization

Brains were harvested and kept in 4% paraformaldehyde and sectioned using a sliding microtome. In situ hybridization was carried out on the tissue sections following the protocol described previously [7]. The primers used for Vglut2 probe were 5’ttggccccgggaaagagg 3’ and 5’agcagtatcgcagccccaaag 3’ [14].

### Statistical Analysis

Statistical analyses were performed using GraphPad Prism 5.0a (GraphPad). Data was analysed by unpaired two-tailed Student’s test or two-way ANOVA followed by Bonferroni’s post hoc tests. The data are represented as mean ± SEM and a *P* value less than 0.05 was considered as statistically significant.

## Results

### Deletion of SF-1 in the VMH Leads to Late Onset Obesity

**Fig 1:**
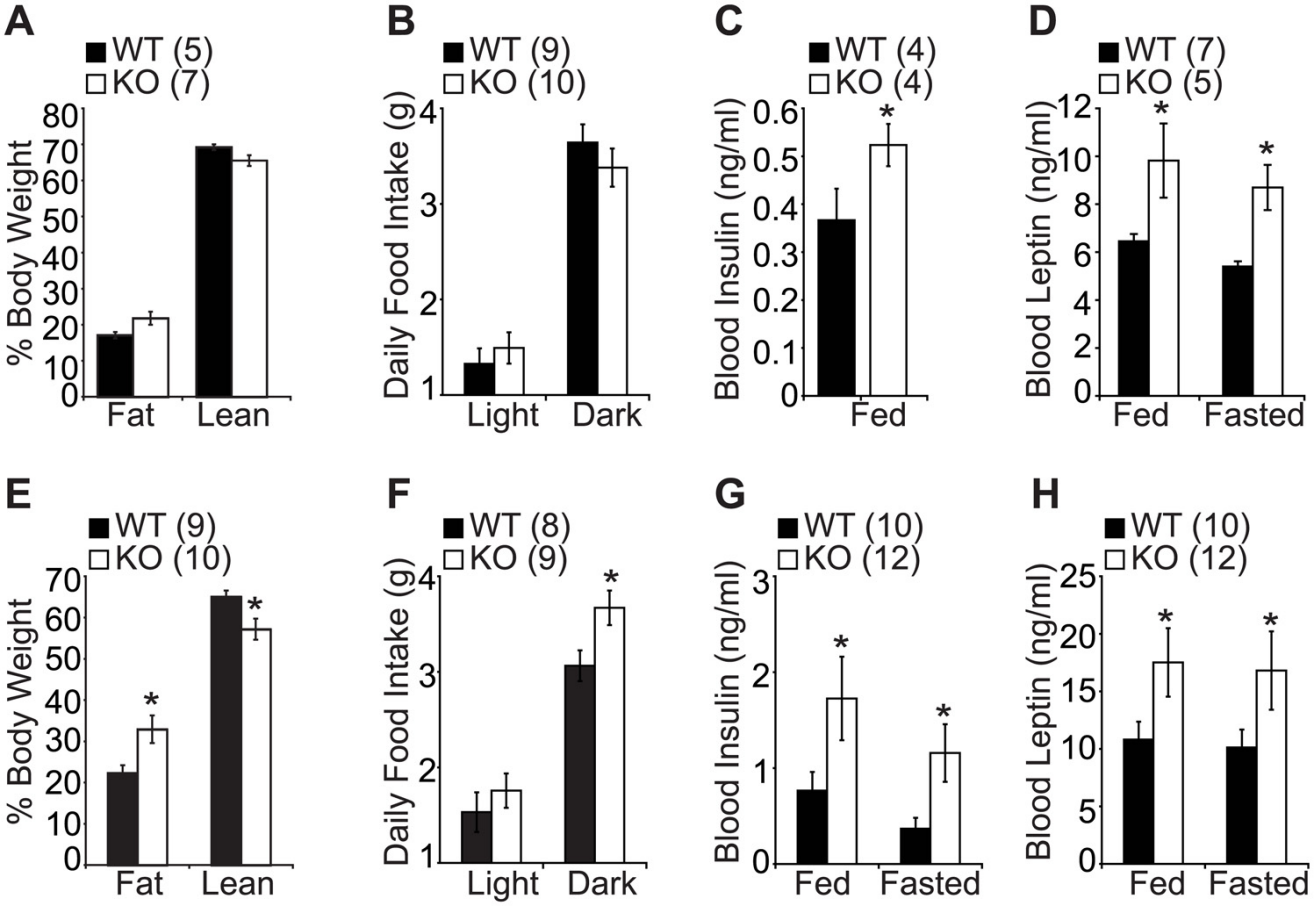
Metabolic phenotypes of young and old VMH-specific SF-1 KO mice. (A and E) percentage body weight, (B and F) daily food intake, (C and G) blood insulin levels, (D and H) blood leptin levels. (A-D) 20–30 weeks old male mice. (E-H) 45–55 weeks old male mice. The number of animals examined is expressed in the parenthesis. The data are represented as mean ± SEM (*P < 0.05, Student’s t-test).

To address the role of SF-1 in the regulation of age-dependent energy homeostasis in the VMH, we generated VMH-specific SF-1 knockout mice (KO) by crossing SF-1 flox mice with SF-1^+/-^ mice expressing nestin-cre as previously described [16]. Metabolic phenotypes were assessed in two different age groups, a young cohort aged 20–30 weeks (young mice) and an old cohort aged 45–55 weeks (old mice). In the young cohort, the WT and KO mice had comparable body weight and fat components (Fig 1A), however in the old cohort, the KO mice showed increased body weight indicated by the significant increase in fat content and marked decrease in lean mass (Fig 1E and S1A Fig). In addition, the daily food intake increased significantly in the old cohort, especially in the dark phase (Fig 1F), but this increase in food intake was not observed in the young cohort (Fig 1B). Interestingly, leptin and insulin were significantly increased in the KO mice in both young and old cohorts (Fig 1C, 1D, 1G and 1H). These results indicate that SF-1 is required for the regulation of normal energy balance in the aging process.

### Regulation of Energy Expenditure by SF-1 in Metabolic Stress Condition

**Fig 2:**
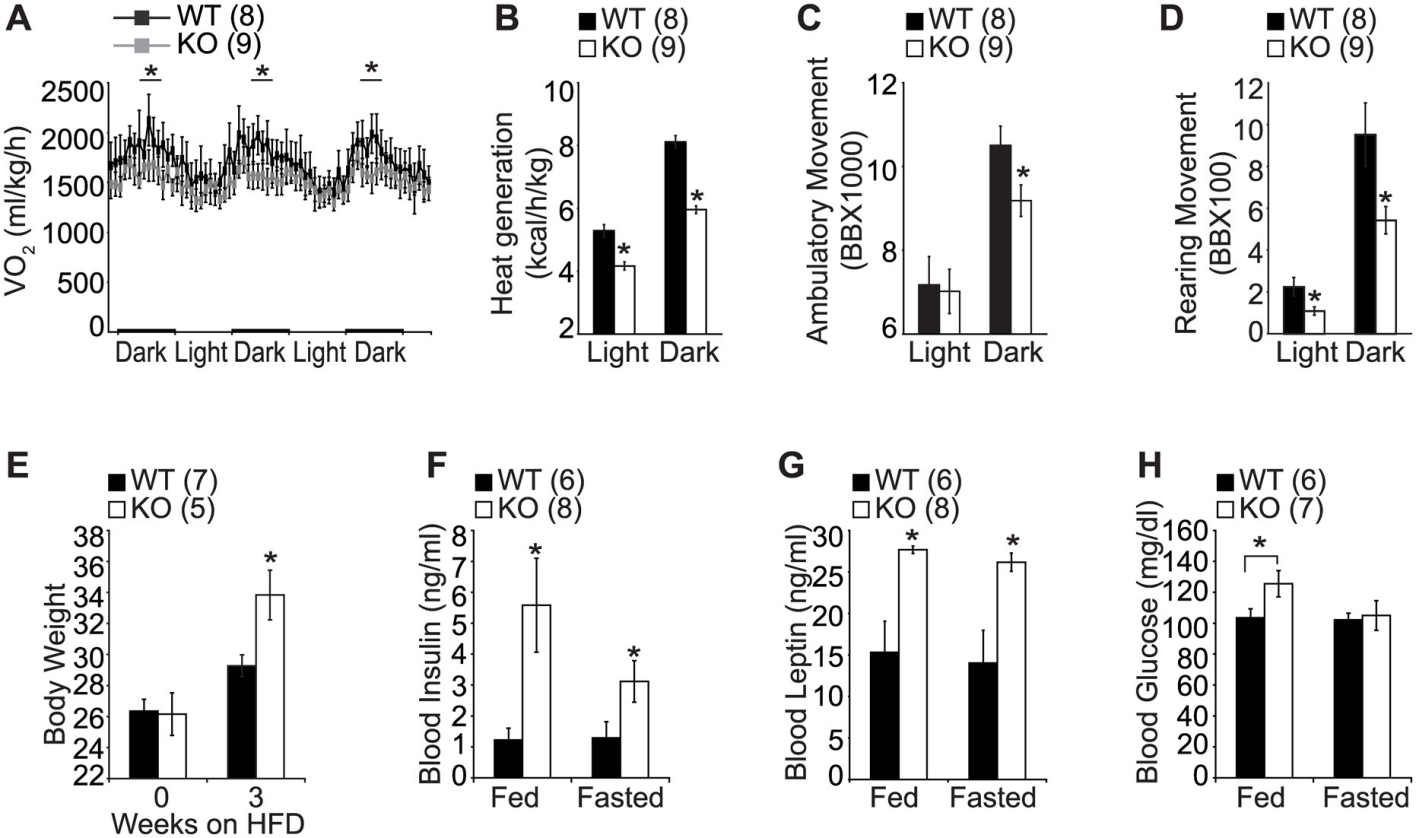
Regulation of energy expenditure by SF-1 in metabolic stress condition. (A) O_2_ consumption, (B) heat generation, (C) ambulatory movement, (D) rearing movement, (E) body weight, (F) blood insulin levels, (G) blood leptin levels, (H) glucose levels. (A-D) 45–55 weeks old male mice. (E-H) 20–30 weeks old male mice challenged with high fat diet for three weeks. Figures in parenthesis indicate number of animals studied. The data are represented as mean ± SEM (*P < 0.05, Student’s *t*-test for bar graphs and two-way ANOVA for comparison of multiple time points). BB, beam break.

To gain mechanistic insight into the deteriorated metabolic profile and hence late onset of obesity in the aged KO mice, we analyzed their metabolic profiles using metabolic chambers. Different from the young cohort (S1B–S1E Fig), the KO mice in the old cohort exhibited a marked reduction in energy expenditure and physical activity, suggesting that the obese phenotype in aged KO mice might be attributed to blunted energy expenditure and locomotor activity together with increased food intake (Figs 1F and 2A–2D). The aging process is accompanied by considerable changes in the efficiency of the metabolic process and aging in this sense could be considered as a metabolic stress condition [17, 18]. We therefore postulated that SF-1 might be essential for maintaining normal body weight homeostasis in different metabolic stress conditions. As high fat diet (HFD) is a well-known metabolic stress condition, besides age, we set out to investigate the role of SF-1 in the regulation of energy homeostasis under HFD. Indeed, HFD exposure resulted in a marked increase in body weight in the KO mice compared to the WT littermates (Fig 2E). In addition, fed and fasted blood insulin and leptin levels and fed glucose levels were significantly elevated in the HFD-fed KO mice compared to WT (Fig 2F–2H).

These results indicate that SF-1 expression in the VMH might be required for the regulation of body weight and hormonal homeostasis in metabolic stress conditions such as aging and high fat diet.

**Fig 3:**
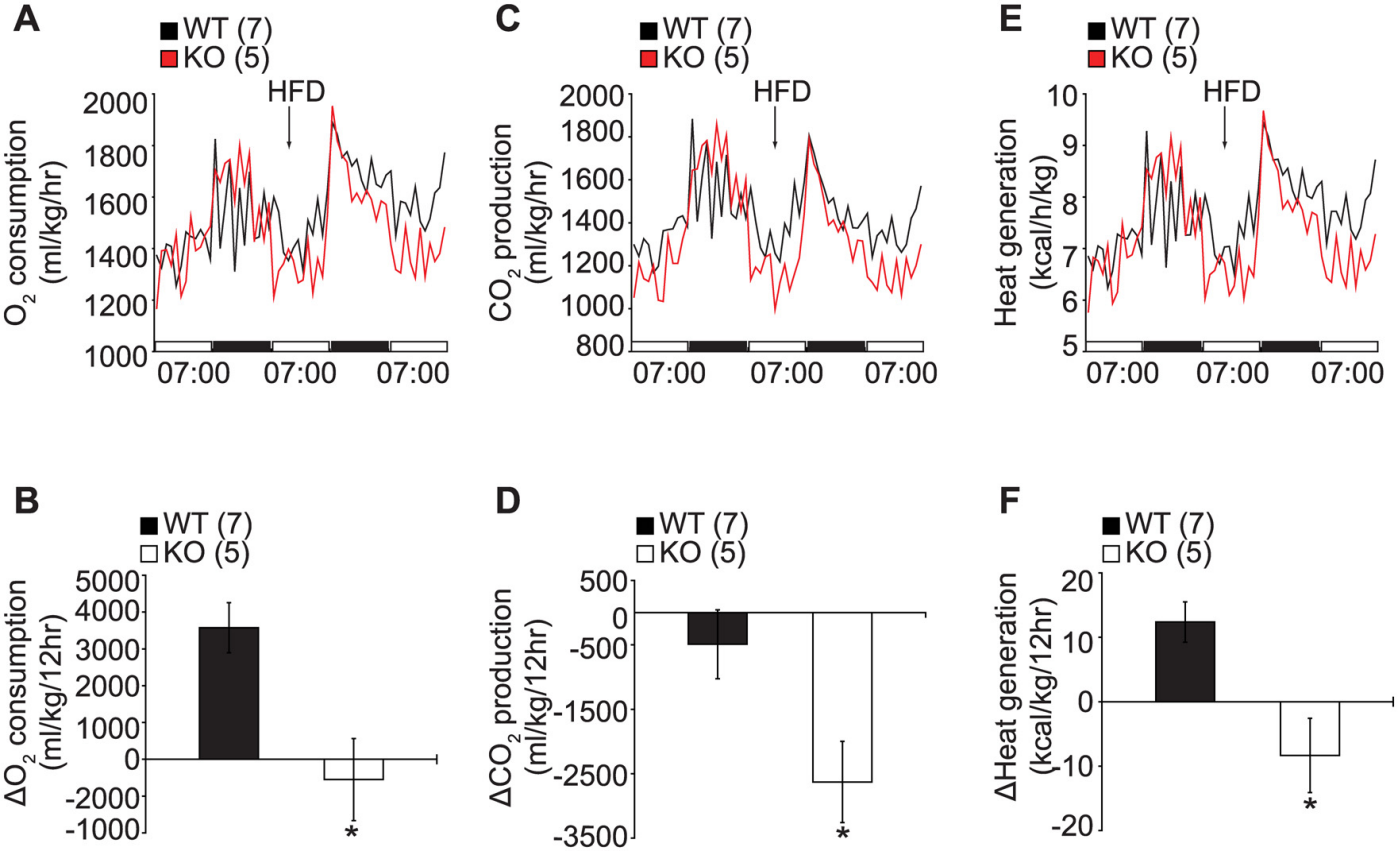
Impaired energy expenditure in SF-1 KO mice under high fat diet. (A and B) O_2_ consumption, (C and D) CO_2_ production, (E and F) heat generation. (A-F) 20–30 weeks old male mice. Figures in parenthesis indicate number of animals studied. The data are represented as mean ± SEM (*P < 0.05, Student’s t-test). HFD, high fat diet.

We next performed metabolic cage studies with mice fed on normal chow diet then switched to high fat diet (HFD) during the study to investigate the underlying mechanism of the diet-induced obesity in KO mice. Switching to HFD induced an increase in energy expenditure in the control mice, however the effect of the HFD on energy expenditure was blunted in the KO mice as evidenced by the marked reduction in oxygen consumption (Fig 3A and 3B), CO_2_ production (Fig 3C and 3D) and heat generation (Fig 3E and 3F) in both the young (Fig 3) and old cohorts (S2 Fig). This data indicates that SF-1 is essential not only for regulation of late onset obesity but also for protection against diet induced obesity.

### Effect of SF-1 Deletion in the VMH on Glucose Metabolism and Insulin Action

**Fig 4:**
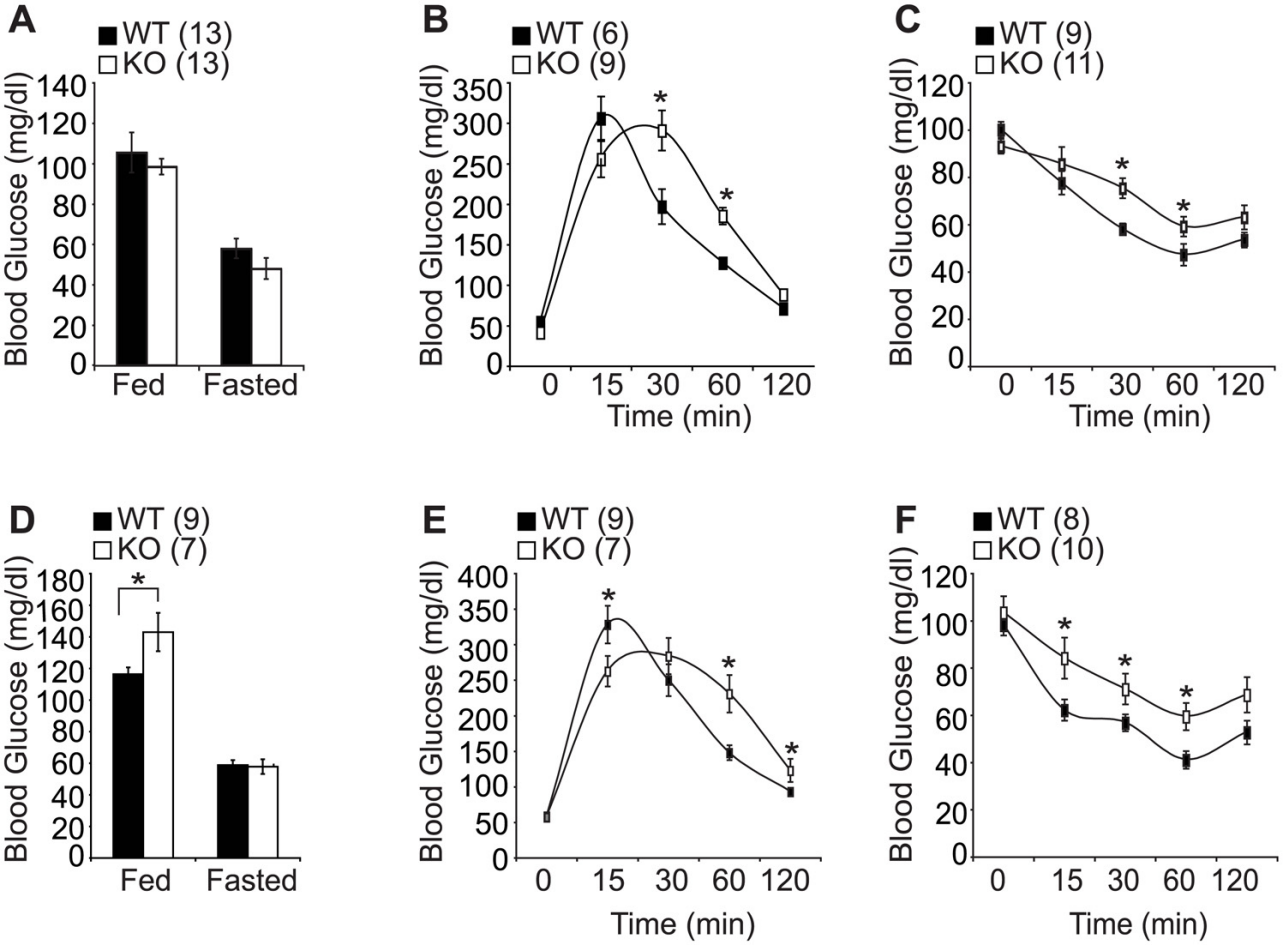
Effect of SF-1 deletion on glucose homeostasis. (A and D) fed and fasted glucose levels, (B and E) glucose tolerance test (GTT), (C and F) insulin tolerance test (ITT). (A-C) 20–30 weeks old female mice. (D-F) 45–55 weeks old female mice. The number of animals examined is expressed in the parenthesis. The data are represented as mean ± SEM (*P < 0.05, Student’s t-test).

Impaired glucose tolerance has been shown to develop with age due to a reduction in glucose induced insulin secretion or due to uncontrolled hepatic glucose output among other factors [19]. Having observed significant insulin and leptin resistance in young SF-1 KO mice (Fig 1C and 1D), we speculated that hormonal dysregulation might already have occurred at a younger age before the development of obesity in aged KO mice. Therefore, we investigated the effect of SF-1 knockout in the VMH on glucose homeostasis and insulin action. We first assayed plasma glucose level at fed and fasted conditions and observed a significant increase in the fed condition only in the old cohort (Fig 4A and 4D). We next performed glucose tolerance tests (GTT) and insulin tolerance tests (ITT) and observed that the KO mice exhibited impaired glucose tolerance (Fig 4B and 4E) and decreased insulin sensitivity (Fig 4C and 4F) in both the young and the old cohorts. These results highly imply that SF-1 is required for normal hormonal regulation from a young age and that the hormonal dysregulation observed in the young mice might contribute, at least partially, to the deteriorated metabolic profile and obese phenotype observed in the aged mice.

### Impaired Thermogenic Gene Expression in BAT and Blunted Vglut2 Expression in SF-1 KO Mice

**Fig 5:**
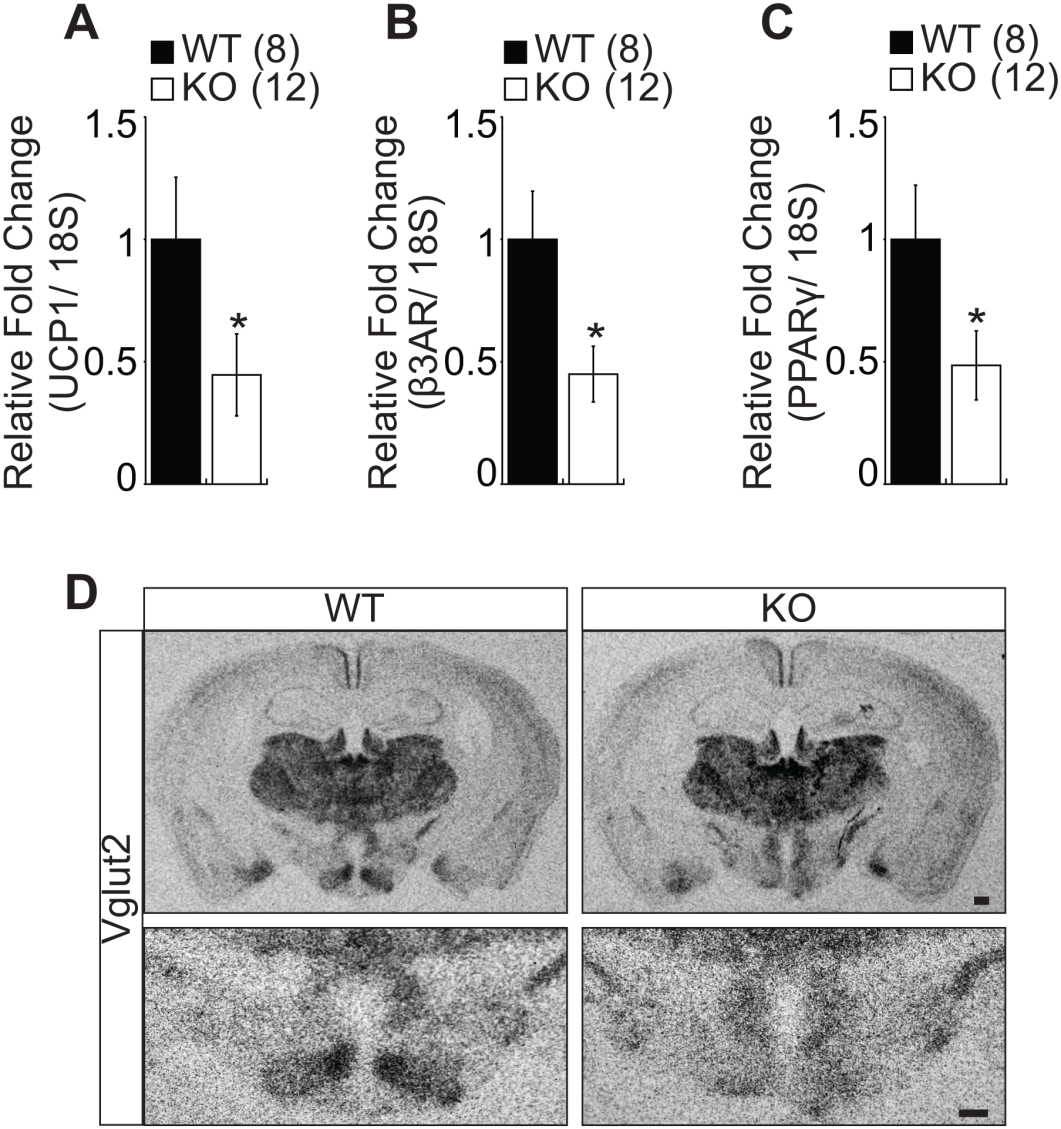
SF-1 deletion leads to impaired gene expression in BAT and blunted Vglut2 expression in the VMH. (A) UCP1, (B) β3AR, and (C) PPARγ expression in brown adipose tissue (BAT) in young male mice (12 weeks). (D) In situ hybridization analyses of Vglut2 expression in the VMH. The number of animals examined is expressed in the parenthesis. Scale bar = 200μm, The data are represented as mean ± SEM (*P < 0.05, Student’s t-test).

The brown adipose tissue (BAT) is known to regulate whole body energy expenditure through sympathetically activated thermogenesis and therefore protect the body against diet-induced obesity [20]. Prompted by the altered energy expenditure in metabolic stress condition including HFD and age in SF-1 KO mice, we sought to explore the molecular mechanism regulating energy expenditure by assaying various genes mediating thermogenesis in the BAT. The KO mice showed significantly reduced levels of uncoupling protein 1 (UCP1), beta 3-adrenergic receptor (β3AR), and peroxisome proliferator-activated receptor γ (PPARγ) indicating that SF-1 might be important for the regulation of thermogenesis through activation of BAT especially in metabolic stress condition including high fat diet and aging (Fig 5A–5C). It has been suggested that the VMH expresses high level of vesicular glutamate transporter 2 (Vglut2) and the deletion of Vglut2 in SF-1 neurons was reported to induce obesity under high fat diet associated with hyperphagia and blunted response to hypoglycemia [14]. In the current study, SF-1 deletion in the VMH also induced hyperphagia, hormonal dysregulation and subsequent obesity development. Therefore, we wondered whether deleting SF-1 in the VMH had any effect on Vglut2 expression. Interestingly, we found a marked reduction in Vglut2 expression specifically in the VMH of SF-1 KO mice (Fig 5D) suggesting that the obese phenotype and hyperphagia observed in SF-1 KO mice might be, at least in part, linked to the change in Vglut2 expression.

## Discussion

Obesity has remained a major public health concern with the prevalence rate rising rapidly over the last few decades. Recent studies have focused on understanding the molecular, environmental and social factors promoting obesity development. A major contributor to the development and pathology of obesity is an imbalance between energy intake and energy expenditure partly due to the modern sedentary lifestyle [21]. The central nervous system (CNS) regulates energy homeostasis by integrating and responding to nutritional signals generated by the peripheral organs such as the circulating insulin and leptin levels. The hypothalamus, particularly the VMH, is a well-known region of the CNS critical for regulating feeding and energy expenditure and therefore maintaining whole body energy homeostasis [22]. In this study, we sought to investigate the mechanism involved in the VMH-mediated energy regulation under different metabolic stress conditions including aging and high fat diet. We took advantage of the Cre-loxP system to delete steroidogenic factor 1 (SF-1) in the VMH using nestin-cre. The VMH-specific SF-1 knockout mice (KO) exhibited late onset of obesity characterized by hyperphagia, hyperinsulinemia, hyperleptinemia and a marked reduction in energy expenditure and physical activity.

Accumulating evidence has shown the significant role played by SF-1 in energy regulation [8–10]. Previous studies have reported that postnatal deletion of SF-1 is accompanied by blunted energy expenditure, however there are no reports describing the age-dependent metabolic roles of SF-1. To explore the importance of SF-1 in the regulation of age-dependent energy metabolism, the metabolic phenotypes of VMH-specific SF-1 KO mice were examined age dependently. In the young cohorts aged 20–30 weeks, the WT and KO mice had comparable fat and lean body mass with no significant change in food intake between the two groups. However in the older cohorts aged 45-55weeks, the KO mice exhibited a marked increase in body weight mainly due to increased food intake and blunted energy expenditure and locomotor activity (Fig 1A, 1B, 1E and 1F). The VMH has been referred to as a glucose sensor as electrical stimulation of VMH neurons was shown to increase glucose uptake in the heart, skeletal muscles, and brown adipose tissues [23]. Similarly, electrolytic destruction of VMH was shown to cause an increase in circulating insulin levels and this hyperinsulinemia was more pronounced following glucose infusion [24]. We found that deletion of SF-1 in the VMH led to an increase in blood insulin and leptin levels in the young and aged mice. (Fig 1C, 1D, 1G and 1H). While the blood glucose level was comparable between the WT and KO mice in the young cohort, glucose and insulin tolerance tests showed that the KO mice had impaired glucose tolerance and reduced insulin sensitivity irrespective of age, suggesting that hormonal dysregulation might have occurred at a young age regardless of obesity onset (Fig 4). In addition, this metabolic disturbance at a young age was exacerbated when the KO mice were exposed to high fat diet indicating the important role played by SF-1 in protection against diet and age induced obesity development (Fig 2E–2H). We postulate that the elevated insulin and leptin levels in SF-1 knockout mice are as a result of the dysregulated energy homeostasis.

A balance between energy consumption and energy expenditure is important for maintaining whole body energy homeostasis. Prompted by the increased food intake and late onset of obesity in the KO mice, we wondered about the effect of SF-1 in the VMH on energy expenditure. Mice were housed in individual metabolic chambers and allowed to acclimatize for 6 days after which their metabolic profiles were assayed for 5 days. In the young cohort, the control and KO mice had comparable oxygen consumption, heat generation, and physical activity (S1 Fig). However, in the old cohort, the KO mice showed a marked reduction in oxygen consumption as well as heat generation. Moreover, the aged KO mice exhibited a reduction in physical activity as shown by the decreased ambulatory and rearing movement especially during the dark phase (Fig 2A–2D). Further, under HFD induced metabolic stress condition, the KO mice showed blunted energy expenditure irrespective of age (Fig 3 and S2 Fig). These data is consistent with previous findings that disruption of the VMH function is accompanied by a reduction in energy expenditure. Specifically, loss of leptin signaling in SF-1 neurons in the VMH resulted in diet induced obesity due to impaired energy expenditure and increased food consumption [8]. Similarly, disruption of the PI3K catalytic subunit, p110α, and postnatal deletion of SF-1 in the VMH also resulted in reduced energy expenditure and development of high-fat diet-induced obesity [10, 15]. These results suggest that SF-1 neurons in the VMH play important roles not only in the regulation of energy intake but also in the modulation of energy expenditure.

The brown adipose tissue (BAT) plays an important role in energy homeostasis by stimulating thermogenesis in response to cold temperatures and by increasing energy expenditure following food intake through the mitochondrial proton carrier, uncoupling protein 1(UCP1) [25]. The BAT activity and subsequent UCP1 activation is mediated by the sympathetic nervous system [26]. In response to activated sympathetic nervous system, norepinephrine is released and binds to β-adrenergic receptors expressed in the BAT cell membrane and this interaction stimulates the release of cyclic AMP (cAMP) and activation of cAMP-dependent protein kinase (PKA) [27, 28] with the subsequent stimulation of UCP1 expression. The nuclear receptor peroxisome proliferator activated receptor γ (PPARγ) is important for the formation and differentiation of the BAT [29] and PPARγ agonists were shown to up-regulate UCP1 expression [30, 31]. In this study we observed blunted energy expenditure specifically in SF-1 KO mice in metabolic stress conditions including HFD and aging. Therefore, we sought to explore the effect of SF-1 deletion in the VMH on BAT activity. We observed a marked down-regulation of UCP1, β3AR, and PPARγ in the BAT of SF-1 KO mice (Fig 5A–5C), highly indicating that SF-1 activity in the VMH might be required for normal BAT function. These findings were supported by previous studies that have reported a link between VMH and BAT activity. For example, lesions in the VMH were reported to disrupt the activity of the sympathetic nervous system by reducing the firing rate of sympathetic nerves to the BAT [32, 33]. These findings indicate that the VMH region as well as the neurons expressed in the VMH play an important role in the SNS activity. We previously reported that deletion of FoxO1 in the SF-1 neurons in the VMH resulted in a lean phenotype due to increased energy expenditure, elevated plasma norepinephrine levels, and increased UCP1 activity in the BAT suggesting that the SF-1 neurons in the VMH may also be involved in the regulation of SNS activity. Although we did not measure the plasma norepinephrine levels in the current study, to partly assess the SNS activity, we expect that deletion of SF-1 in the VMH is likely to be associated with decreased norepinephrine levels as shown by the diminished BAT activity and the late-onset obesity. It would therefore be interesting to carry out further studies and examine if SF-1 in the VMH is directly linked to BAT activity through the regulation of sympathetic nervous system.

The VMH is considered to be largely glutamatergic as indicated by the high expression of vesicular glutamate transporter 2 (Vglut2) [34] which is important for glutamate uptake into synaptic vesicles of excitatory neurons. Deletion of Vglut2 in SF-1 neurons in the VMH was reported to cause obesity under high fat diet associated with hyperphagia and increased adiposity accompanied by hypoglycemia specifically in fasted condition, indicating that Vglut2 plays a role in regulating energy balance in the VMH [14]. We observed a marked reduction in the expression of Vglut2 in the VMH of the KO mice (Fig 5D) indicating that the metabolic phenotype observed in SF-1 KO mice might be, at least in part, as a result of diminished Vglut2 expression.

The metabolic phenotype observed in aged SF-1 KO mice including increased body weight, fat mass, and food intake accompanied by a reduction in energy expenditure and physical activity implies that SF-1 is essential for the regulation of age-dependent energy metabolism. In addition, the dysregulation of leptin and insulin homeostasis from a young age and in different dietary conditions indicates that SF-1 is required for metabolic homeostasis and that SF-1 might protect the body against metabolic stresses induced by high fat diet and aging. Collectively, our findings point to the important role of SF-1 in the regulation of age-dependent metabolic diseases.

## Supporting Information

S1 Fig Metabolic phenotype of SF-1 KO mice. (A) Representative images depicting the difference in body weight between aged WT and KO mice. (B) Oxygen consumption, (C) heat generation, (D) ambulatory movement, and (E) rearing movement from 20–30 weeks old male mice. The number of animals examined is expressed in the parenthesis. The data are represented as mean ± SEM (*P < 0.05, Student’s t-test).
(EPS)

S2 Fig Impaired energy expenditure in aged SF-1 KO mice exposed to HFD. (A and B) O_2_ consumption, (C and D) CO_2_ production, (E and F) heat generation from male mice aged 45–55 weeks. The number of animals examined is expressed in the parenthesis. The data are represented as mean ± SEM (*P < 0.05, Student’s t-test).
(EPS)
